# The role of effort-reward imbalance and depressive symptoms in the relationship between long working hours and presenteeism among Chinese village doctors: a moderated mediation model

**DOI:** 10.1186/s12888-023-04986-4

**Published:** 2023-07-11

**Authors:** Jingya Ji, Yarong Han, Ruyu Li, Hui Jin, Chengjie Yin, Luyao Niu, Xinyu Ying, Yuexia Gao, Qiang Ma

**Affiliations:** 1 Department of Health Management, School of Public Health, Nantong University, 9 Seyuan Road, Jiangsu, 226019 China; 2 Department of Health Management, Affiliated Hospital of Nantong University, Nantong, China; 3 Nantong Center for Disease Control and Prevention, Nantong, China; 4 Institute for Health and Development, Nantong University, Nantong, China

**Keywords:** Presenteeism, Long working hours, Effort-reward imbalance, Depressive symptoms, Village doctors

## Abstract

**Background:**

Long working hours and effort-reward imbalance (ERI) among medical workers may contribute to poor mental health and reduced productivity. However, the potential mechanisms among them are not well understood. This study aimed to explore the role of depressive symptoms and ERI in the relationship between long working hours and presenteeism among village doctors.

**Methods:**

We conducted a cross-sectional study in Jiangsu Province, eastern China. 705 village doctors were assessed for working hours, ERI (the Effort-Reward Imbalance Questionnaire, ERI questionnaire), presenteeism (6-item Stanford Presenteeism Scale, SPS-6 Scale), and depressive symptoms (12-item General Health Questionnaire, GHQ-12). A moderated mediation model was used to test the role of depressive symptoms (M), and ERI (W) in the relationship between long working hours (X) and presenteeism (Y).

**Results:**

45.11% of the village doctors worked more than 55 h per week, and 55.89% were exposed to ERI. The prevalence of depressive symptoms among Chinese village doctors was 40.85%. Long working hours (≥ 55 h per week) were significantly associated with presenteeism behaviors (β = 2.17, *P* < 0.001). Mediation analysis demonstrated that depressive symptoms (GHQ score > 3) could partly mediate the relationship between long working hours and presenteeism (indirect effect β = 0.64, *P* < 0.001). Moderated mediation further indicated that the interaction of long working hours and ERI was significantly and positively associated with depressive symptoms, which in turn predicted elevated presenteeism behaviors.

**Conclusions:**

Depressive symptoms had a mediating role in the association of long working hours with presenteeism behaviors among Chinese village doctors and ERI augment their negative effects.

## Introduction

Presenteeism has been characterized in various ways as it develops conceptually. Its definition has been expanded to the behavior leading to recessive lost productivity or work ability due to health problems or other factors [1–4], instead of only focusing on sickness presenteeism. Studies have demonstrated that presenteeism is highly significant due to its detrimental effect on both individual health and organizational productivity [5, 6]. About 60% of employees in the United States and over 70% of Danish employees have experienced presenteeism [7, 8]. Specifically, medical staff faced different stresses and pressures due to presenteeism, like heavy workloads and difficulties finding a substitute due to a labor shortage, as well as organizational culture hurdles and professional standards against taking sick leave [9–11]. Village doctors, who safeguard health in rural China, are playing a key role in the healthcare system. China built its rural primary health care system in the 1960 and 1970 s, which included “barefoot doctors,“ the (old) cooperative medical scheme, and a three-tiered service delivery system at the county, township, and village levels [12]. Peasants who got only rudimentary medical and paramedical training and served in rural villages in China were known as barefoot doctors. In the early 1980s, China abandoned the name “barefoot doctor” in favor of “village doctor.“ As specialized medical workers, village doctors are sometimes required to work in solitude, with minimal support, feedback, or oversight from others [13]. Adverse working conditions have a substantial effect on employee health and well-being and reduce productivity owing to absenteeism and presenteeism [14]. Thus, combating presenteeism may be a key to improving village doctors’ health productivity.

The job-demand-resource (JD-R) theory [15] has been widely adopted to investigate the association between job demands that function as stressors and are hazardous to health and the resources that counter the stress effects of job demands. Job demand is defined as those physical, psychological, social, or organizational aspects of the job that require sustained effort, which creates job burnout, presenteeism, and depression [16]. Long working hours are thought to be the main indicator of presenteeism. Working long hours, frequently as a result of heavy workloads, increases the risk of working when sick to avoid the accumulation of work responsibilities [17]. Long working hours and high working intensity make occupational work and rest irregular. One study conducted in Korea found that those who worked more than 60 h per week had the greatest percentage of presenteeism [18]. There was little research conducted on Chinese village doctors, nevertheless. Thus, we proposed hypothesis 1: the longer the village doctors worked, the more presenteeism the village doctors would perform.

Long working hours may contribute to depression or depressive symptoms in village doctors, which may further lead to presenteeism. Working for an extended amount of time requires prolonged and energy-draining efforts that may exhaust an individual’s resources and damage their well-being [19]. And having to work for a long time would deprive employees of the chance to rest and replenish energy. Such constant depletion of the resource increases the risk of both physical and mental sickness [20]. Most studies have demonstrated that depression increases sickness absence, but few studies have shown that depressive symptoms impair an employee’s capacity to function adequately at work, a phenomenon known as presenteeism. Medical workers are particularly vulnerable to experiencing mental health problems due to heavy workloads and high levels of work-related stress [21]. Yet, little is known about depressive symptoms among Chinese village doctors. So we proposed the following hypothesis: depressive symptoms may mediate the relationship between long working hours and presenteeism (Hypothesis 2).

Siegrist and colleagues developed the effort-reward imbalance (ERI) model in the field of sociology in 1996 [22]. This model’s theoretical foundation holds that employees’ efforts at work are one aspect of a reciprocal agreement in which they are compensated (monetary reward, respect, and status) in exchange for those efforts. When the required job effort is perceived as difficult while the associated benefits are meager, the resultant imbalance might result in potential pathological consequences, either physical or mental [23]. Several studies have been conducted on employee recognition in the workplace and its effect on physical and psychological health, but few studies have been conducted on the association of employee recognition on presenteeism. In a study conducted in Michigan among hotel housekeepers, higher ERI has been linked to higher presenteeism and work productivity [24]. However, there was little research conducted on medical workers, particularly Chinese village doctors. Hence, we proposed the hypothesis as follows: ERI can moderate the relationship between long working hours and presenteeism through depressive symptoms (Hypothesis 3).

**Fig. 1:**
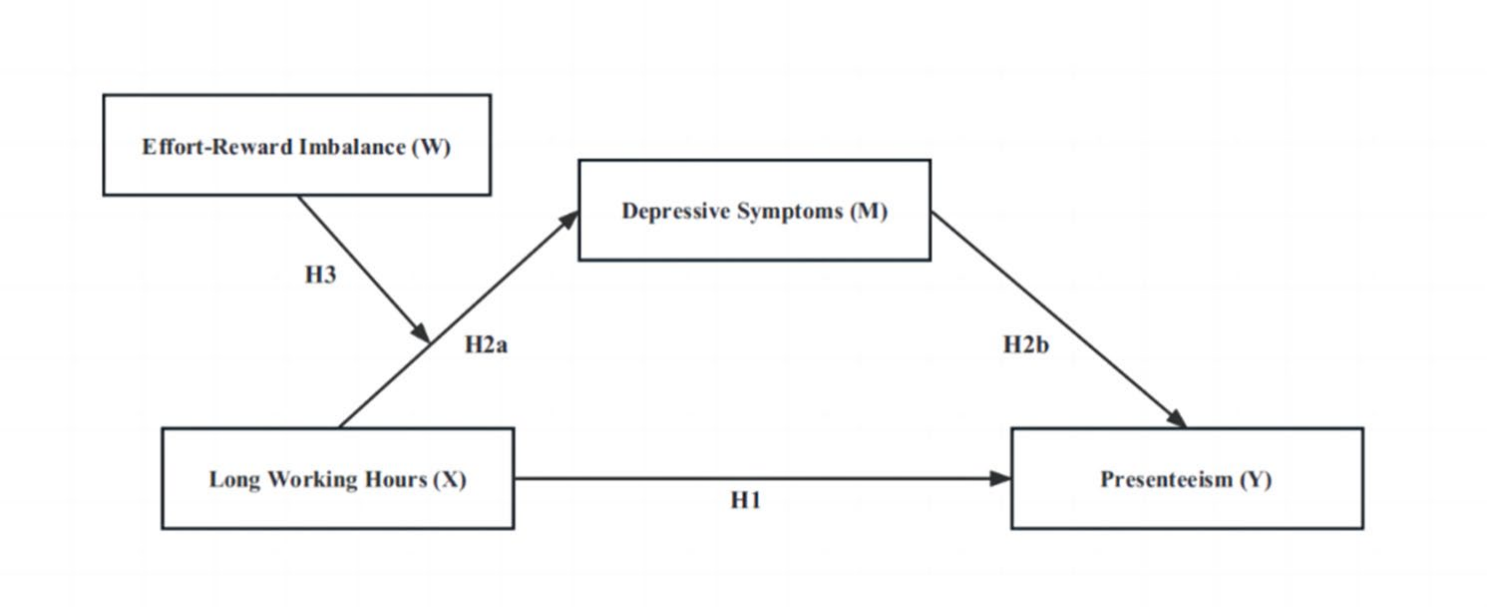
The proposed conceptual framework indicating the relationships between long working hours, effort-reward imbalance, depressive symptoms and presenteeism

In general, long working hours, ERI, depressive symptoms, and presenteeism are all correlated. Yet, to date, few studies have examined the potential links among these variables, especially among village doctors in rural China. Therefore, based on the JD-R model and ERI model, this study proposed the three hypotheses (Fig. 1) and employed moderated mediation models to examine the associations among these variables.

## Materials and methods

### Study design and setting

This cross-sectional study was conducted from May to October 2019 in three districts (Tongzhou district and Haimen district in Nantong city, Dafeng district in Yancheng city) in Jiangsu Province of eastern China, where were districts with an aging population and high chronic non-communicable diseases (NCDs) burden. A total of 750 village doctors who attended township health center meetings were invited for the survey and 705 (284 males, 421 females) valid questionnaires were collected (response rate = 94.0%). Any questionnaire with a completion rate of 90% was regarded as efficient.

Village doctors working in rural clinics responsible for NCDs management will be selected and surveyed. Target population eligible for this study should comply with all of the following inclusion criteria: (i) had a practicing qualification certificate from the local health administration departments at or above the county level; (ii) had a work tenure of more than 1 year; (iii) provided managed-care for at least 50 NCDs patients. 750 participants were informed and completed a face-to-face interview for about ten minutes. When finished the interview, they were given a randomized red envelope from 20 to 50 RMB (2.80$ to 7.00$).

This study was approved by the Ethics Committees of Nantong University, and informed consent was obtained from each participant.

### Measurements and variables

#### Control variables

Demographic variables included age, gender, education (divided as technical secondary school and junior college or above), work tenure, and contract status (trichotomized as permanent, temporary, and other).

#### Working hours

Working hours were estimated by the following questions “In the past two weeks, how many hours did you work on an average day? How many days a week did you work (including weekdays and weekends, ranging from 1 to 7 )?” Working hours were rounded up to the nearest hour. Based on a previous study [25], the calculation method was as follows:

Mean weekly working hours = (mean working hours per day) × (working days).

According to the working hours per week, working hours were categorized as: <55 h and ≥ 55 h. As for the categorization, in the majority of cohorts, long working hours was defined as working ≥ 55 h per week [26].

#### Effort-reward imbalance

The Effort-Reward Imbalance questionnaire(the ERI questionnaire) was adapted to assess occupational stress, which was developed by Siegrist and colleagues in 1996 and compiled in 2006 by Li Xiuyang. This instrument has 23 items that are distributed in three scales: effort (6 items), reward (11 items), and over-commitment (6 items) [22, 27]. ERI items were scored on a 5-point Likert score, ranging from 1 “strongly disagree” to 5 “strongly agree.” The scores of each item were added to obtain a three-dimension score, and the higher the score, the higher the level of the respective dimension. The ERI ratio was calculated as follows:

ERI = effort/reward × correction factor (factor correcting for the difference in the numbers of items of the two scales; in this analysis, correction factor = 6/11).

The ratio score exceeding 1.0 was considered to reflect a high-risk imbalance condition for high effort and low reward. In this study, the internal consistency reliability of Cronbach’s alphas of the three dimensions in the ERI scale was 0.91, 0.74, and 0.84, respectively. The measure showed good internal consistency.

#### Depressive symptoms

The Chinese version 12-item General Health Questionnaire (GHQ-12) was used to evaluate depressive symptoms [28]. For each item, the participants were asked to select a response from a list of options to describe particular symptoms and whether their emotional state differed from their regular condition with the options as follows: much less than usual, same as usual, rather more than usual, and much more than usual. All items were coded using a 0-0-1-1 pattern such that “less” and “usual” responses were coded as 0, and “worse” and “much worse” responses were coded as 1. The total scores are the sum of positive and negative, varying from 0 to 12. The higher scores indicated more severe mental illnesses. A score exceeding 3.0 was considered to indicate depressive symptoms. The GHQ-12 has shown high reliability and good sensitivity (α = 0.91) in our study.

#### Presenteeism

Presenteeism was assessed by the 6-item Stanford Presenteeism Scale (SPS-6) [29]. This instrument is distributed in two scales: completing work (focus work outcome) and avoiding distraction (focus work processes). The SPS-6 follows a 5-point Likert scale, ranging from 1 “strongly disagree” to 5 “strongly agree”. The SPS-6 score is the sum of positive and reverse scores, ranging from 6 to 30, with a higher score indicating greater productivity loss caused by presenteeism. SPS-6 has shown high reliability and validity in the Chinese professional population (α = 0.87). In this survey, the Cronbach’s coefficient of the scale was 0.82.

### Statistical analysis

Statistical analyses were conducted using SPSS 26.0 and Hayes SPSS macro program PROCESS. First, the basic characteristics of participants were described using summary statistics, that is, mean, standard deviations (continuous variables), frequency distribution and percentage (categorical variables). Partial correlations analysis was calculated to examine correlations between the independent variable (X), mediator (M), moderator (W) and dependent variable (Y). Second, the model 4 of the PROCESS was used to test the relationship between the long working hours (X) and presenteeism (Y) and the mediating role of depressive symptoms (M) among this relationship. Model 1 examined the relationship between the long working hours (X) and presenteeism (Y) (H1 in Fig. 1). Model 2 tested the relationship between the long working hours (X) and depressive symptoms (M) (H2a in Fig. 1) and model 3 tested the direct effect of the long working hours (X), depressive symptoms (M) on presenteeism (Y) (H2b in Fig. 1). These models provided direct and indirect paths among these variables. The details for model 4 of the PROCESS have been presented in Table 1.

**Table 1:** Mediation models for long working hours on presenteeism through depressive symptoms

Variables	Presenteeism score		GHQ score		Presenteeism score
	Model 1		Model 2		Model 3
**Control variables**					
Age	0.00(0.04)		-0.06(0.02)^*^		0.04(0.03)
Gender	-0.92(0.34)^**^		-0.55(0.22)^**^		-0.60(0.32)
Education	-0.97(0.39)^**^		-0.68(0.24)^*^		-0.57(0.36)
Work tenure	0.01(0.03)		0.05(0.02)^*^		-0.02(0.03)
Contract status	0.31(0.22)		0.01(0.14)		0.30(0.20)
**Independent variable**					
Working hours	2.17(0.33)^***^		1.08(0.21)^***^		1.52(0.32)^***^
**Mediator**					
GHQ score	--		--		0.59(0.06)^***^
Constant	18.69(1.49)^***^		6.64(0.94)^***^		14.75(1.43)^***^
Adj-R^2^	0.14		0.10		0.26
F	19.62^***^		13.02^***^		35.70^***^

Note: The parentheses outside are the β values and the parentheses inside are the standard errors of the β values. Gender: male = 0, female = 1; Education: technical secondary school = 0, junior college or above = 1; Contract status: permanent = 0, temporary = 1; Other = 2; Working hours: <55 h = 0, ≥55 h = 1. * *P* < 0.05, ** *P* < 0.01, *** *P* < 0.001

**Fig. 2:**
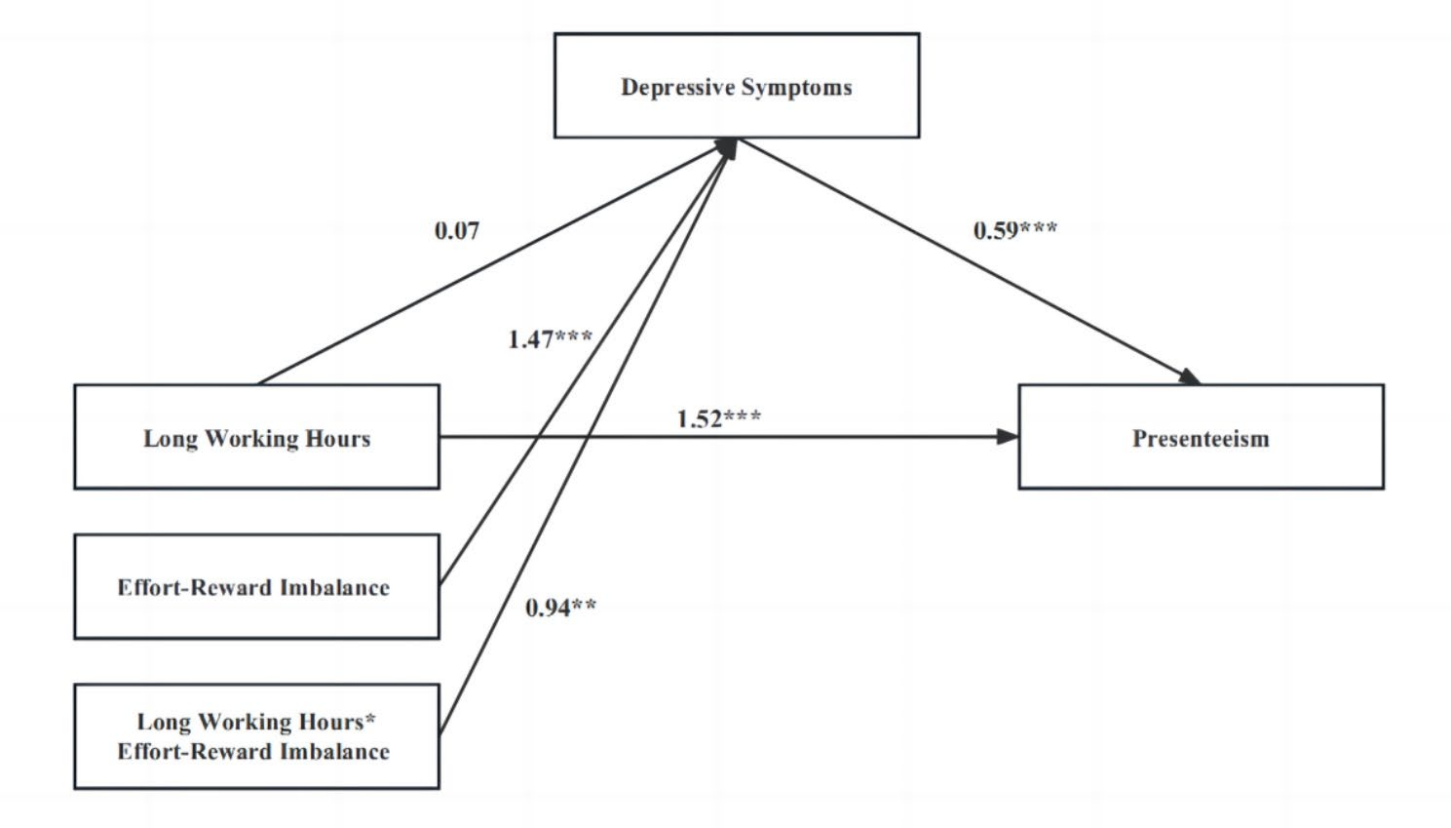
Results for the testing hypothesis. * *P* < 0.05, ** *P* < 0.01, *** *P* < 0.001. Covariates: age, gender, education, work tenure, and contract status

**Fig. 3:**
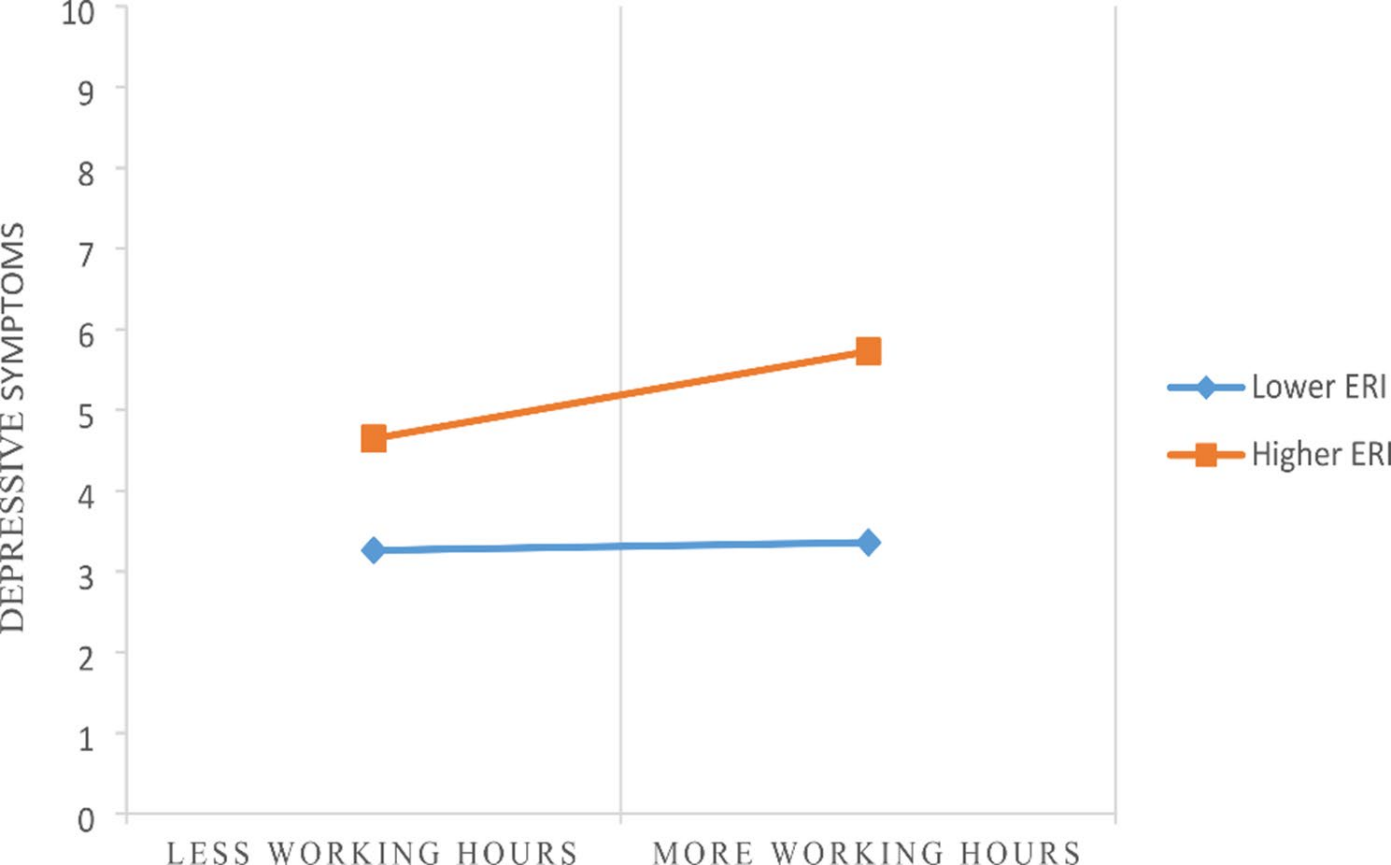
The moderating effect of ERI on the relationship between working hours and depressive symptoms

Third, the proposed moderator variables of ERI (W) were were integrated into the model for testing the moderated mediation hypothesis (Hypothesis 3) using model 7 of the PROCESS. The moderating effect of ERI (W) in the relation of long working hours to depressive symptoms would be examined (H3 in Fig. 1) in Model 4a. If the coefficients of the interaction of ERI (W) and working hours were significant, indicating ERI strengthened the Chinese village doctors from the negative effect by increasing their depressive symptoms. The details for model 7 have been presented in Table 2; Fig. 2, the value of moderated indirect effects were calculated and presented in Table 3. The simple slope test was conducted to describe the moderating effect of ERI in Fig. 3. All the control variables were entered in all analyses. Both the mediation and moderated mediation analysis was performed based on 5,000 bootstrapped samples and bias-corrected 95% confidence intervals (CI) were calculated. If the confidence interval values do not contain zero, the mediating and moderated mediating effects can be considered significant.

**Table 2:** Moderated mediation regressions of long working hours,ERI, depressive symptoms, and presenteeism

Variables	GHQ score		Presenteeism score
	Model 4a		Model 4b
**Control variables**			
Age	-0.06(0.02)^*^		0.04(0.03)
Gender	-0.36(0.20)		-0.60(0.32)
Education	-0.64(0.23)^*^		-0.57(0.36)
Work tenure	0.05(0.02)^*^		-0.02(0.03)
Contract status	0.02(0.13)		0.30(0.20)
**Independent variable**			
Working hours	0.07(0.31)		1.52(0.32)^***^
**Mediator**			
GHQ score	--		0.59(0.06)^***^
**Moderator**			
ERI	1.47(0.25)^***^		--
**Interaction**			
Working hours*ERI	0.94(0.39)^**^		--
Constant	5.61(0.89)^***^		14.75(1.43)^***^
Adj-R^2^	0.21		0.26
F	23.63^***^		35.70^***^

Note: The parentheses outside are the β values and the parentheses inside are the standard errors of the β values. Gender: male = 0, female = 1; Education: technical secondary school = 0, junior college or above = 1; Contract status: permanent = 0, temporary = 1; Other = 2. * *P* < 0.05, ** *P* < 0.01, *** *P* < 0.001

**Table 3:** Conditional indirect effects of the long working hours on presenteeism through depressive symptoms at different levels of ERI

Groups	Effect	BootSE	BootLLCI	BootULCI
Low level of ERI (M-1SD)	0.04	0.15	-0.25	0.33
High level of ERI (M + 1SD)	0.60	0.18	0.26	0.98

Note: BootLLCI = Bootstrap confidence intervals with lower limits; BootLLCI = Bootstrap confidence intervals with upper limits

### Common method bias

The psychological and work-related data were collected simultaneously through self-rating questionnaire, thus common method bias (CMB) was a potential problem. In order to minimize this effect as much as possible, we controlled from the procedures, such as anonymous testing, careful questionnaire arrangement, etc. Meanwhile, Harman’ s one-factor test was applied to test for the CMB [30]. The first factor explains variance less than 50%, suggesting that common method variance does not represent a problem in the study. Principal component analysis with varimax rotation results showed that the first factor accounted for 28.291% of the variance, indicating CMB was not significant in our study.

## Results

### Participant characteristics

705 participants comprised more women (59.72%) and were not highly educated (54.61% had a technical secondary school qualification), with a mean age of 45.25 years (SD 12.36). Almost half (45.11%) of them worked more than 55 h per week, and 55.89% were exposed to ERI. The mean score of presenteeism was 17.67 (SD 4.40). The prevalence of depressive symptoms was 40.85%. The ERI differences in gender, age, education, work tenure, working hours, GHQ score, depressive symptoms, and presenteeism score were statistically significant (see in Table 4).

**Table 4:** Basic characteristics of the participants (N = 705)

Variables	N (%)	ERI ≤ 1	ERI > 1	χ^2^/F	*P*
		(N = 311)	(N = 394)		
Gender					
Male	284(40.28)	99(31.83)	185(46.95)	16.52	< 0.001
Female	421(59.72)	212(68.17)	209(53.05)		
Age(years)(mean ± SD)	45.25 ± 12.36	43.38 ± 13.18	46.73 ± 11.47	13.01	< 0.001
Education					
Technical secondary school	309(43.83)	115(36.98)	194(49.24)	10.61	0.001
Junior college or above	396(56.17)	196(63.02)	200(50.76)		
Work tenure(years)(mean ± SD)	23.52 ± 12.90	21.59 ± 13.81	25.05 ± 11.93	12.65	< 0.001
Contract status					
Permanent	411 (58.30)	181(58.20)	230(58.38)	5.34	0.069
Temporary	189 (26.81)	93(29.90)	96(24.37)		
Other^a^	105 (14.89)	37(11.90)	68(17.26)		
Working hours(hours)(mean ± SD)	55.32 ± 14.42	50.55 ± 12.15	59.09 ± 14.95	66.73	< 0.001
Working hours (hours)					
<55	387 (54.89)	218(70.10)	169(42.89)	51.94	< 0.001
≥ 55	318 (45.11)	93(29.90)	225(57.11)		
GHQ score(mean ± SD)	3.87 ± 2.70	2.67 ± 1.76	4.81 ± 2.93	129.69	< 0.001
Depressive symptoms(GHQ > 3)	288 (40.85)	71(22.83)	217(55.08)	74.80	< 0.001
Presenteeism score(mean ± SD)	17.67 ± 4.40	15.58 ± 4.39	19.31 ± 3.64	151.77	< 0.001

Note: ^a^ retired personnel who return to work

### Correlations among variables

Partial correlations of the main variables were shown in Table 5. Working hours and ERI were significantly and positively correlated with both depressive symptoms and presenteeism. Depressive symptoms were also positively correlated with presenteeism (r = 0.44, *P* < 0.001).

**Table 5:** Partial correlations among working hours, ERI ,depressive symptoms and presenteeism

Variables	Mean ± SD	Working hours	ERI	Presenteeism	Depressive symptoms
Working hours	55.32 ± 14.42	1			
ERI	1.05 ± 0.30	0.31***	1		
Presenteeism	17.67 ± 4.40	0.29***	0.43***	1	
Depressive symptoms	3.87 ± 2.70	0.31***	0.47***	0.40***	1

Note: This model controlled for demographic variables, including age, gender, education, work tenure, and contract status. *P < 0.05, **P < 0.01, *** *P* < 0.001

### Depressive symptoms as mediator

The mediation regression results indicated that long working hours was significantly and positively related to presenteeism (β = 2.17, *P* < 0.001) after adjusting for covariates (see Model 1 in Table 1), providing support for Hypothesis 1. Working longer hours significantly predicted higher depressive symptoms (β = 1.08, *P* < 0.001) (see Model 2 in Table 1), which, in turn, was significantly associated with high presenteeism score (β = 0.59, *P* < 0.001) (see Model 3 in Table 1). When depressive symptoms entered in the equation model, the regression coefficient for presenteeism concerning long working hours (from β = 2.17 to β = 1.52, *P* < 0.001) reduced and was still significant, indicating that depressive symptoms partially mediate the relationship between long working hours and presenteeism.

### ERI as moderator

Moderated mediation regression results were shown in Table 2; Fig. 2. As the moderator of ERI and the interaction terms were entered into the models, long working hours did not significantly predict depressive symptoms (β = 0.07, *P >* 0.05), but the moderator of ERI (β = 1.47, *P <* 0.001) and the interaction of ERI and long working hours (β = 0.94, *P <* 0.01) significantly predict depressive symptoms, which indicated that ERI strengthened the detrimental effect of long working hours on depressive symptoms, providing support to hypothesis 3. Specifically, Fig. 3 showed the conditional direct effects of depressive symptoms (M) on presenteesim (Y) at different moderator levels, where there were stronger direct effect in the higher level of ERI than lower level ERI.

The value of moderated mediating effects were calculated and results in Table 3 further verified that the indirect effects of the long working hours on presenteeism through depressive symptoms were moderated by ERI. The indirect effect was significant for participants with high level ERI (estimated effect = 0.60, 95% CI [0.26, 0.98]), whereas this indirect effect was not significant for participants with low level ERI (estimated effect = 0.04, 95% CI [− 0.25, 0.33]).These results highlighted that in the presence of high level ERI, the effects of long working hours on presenteeism through depressive symptoms strengthened.

Additionally, we test four continuous variables (long working hours, ERI score, GHQ score, and presenteeism score) in the same statistical analysis, and the results are consistent with the above results.

## Discussion

In this study, the mediating role of depressive symptoms and the moderating role of ERI in the relationships between long working hours and presenteeism were identified among Chinese village doctors. Based on the JD-R model and Effort-reward Imbalance model, our study hypothesized the negative effect of work-related demands on presenteeism and employed moderated mediation analysis to test the hypothesis and reveal several findings. First, long working hours had a direct positive relationship with the presenteeism of Chinese village doctors. Meanwhile, depressive symptoms, as an independent mediator, significantly mediated the effect of long working hours on presenteeism. Moreover, ERI significantly moderated the detrimental effect of long working hours on depressive symptoms. Fourth, ERI strengthened the mediating pathways of long working hours on presenteeism through depressive symptoms. The current findings, in general, coincide with those reported previously: long working hours and ERI may contribute to the feeling of humiliation and deteriorate subjective well-being, and these are considered essential psychological processes in the development of depressive symptoms [31]. These health conditions are associated with presenteeism [32] and loss of productivity [33].

A meaningful finding of our study is that long working hours are positively related to presenteeism among Chinese village doctors, confirming the findings of previous meta-analyses [34, 35]. According to a Korean study, working long hours was a significant risk factor for presenteeism [18]. The substantial association between working hours and presenteeism can be explained by the negative health effects of long working hours. As workload and stress increased, village doctors were more likely to compare their efforts and rewards. When they found that their efforts did not match their rewards, the doctors were less engaged in their work and exhibited negative behaviors such as job burnout and presenteeism. Thus, further investigating whether and how working hours affect village doctors’ work status would improve our understanding of the risk of long working hours.

The main contribution of our research is that it demonstrated the robust mediating role of depressive symptoms in the associations between long working hours and presenteeism, which provides evidence for understanding the detrimental effect of long working hours on mental health and work status. Virtanen et al. reported that individuals working long hours in Asian countries had a 1.5-fold increased risk of depressive symptoms during follow-up [36]. Despite the fact that few studies have investigated the impact of depressive symptoms on presenteeism, depressive symptoms have been related to several negative work outcomes, such as work loss and work cutback [37–39] and job turnover [40]. These negative work outcomes suggest a reduction in work abilities and performance. The present study defined presenteeism from the perspective of productivity loss; thus, people with severe depressive symptoms might have higher presenteeism. Furthermore, Jain et al. [41] examined full-time employees diagnosed with depression and observed a drop in overall productivity at all levels of depression and a worsening of presenteeism and absenteeism as the degree of depression increased. In China, village doctors, who usually have low incomes, heavy workloads, and limited medical knowledge, are considered to have a higher risk of stress and depressive symptoms. Aging and a lack of financial incentives and career advancement are serious issues for primary care workers, especially in rural China, which would decrease productivity and increase presenteeism among village doctors.

Another interesting contribution of our research is that we conducted a moderated mediation model to explore the moderating role of ERI and interactions between job demands and depressive symptoms in the relationship between long working hours and presenteeism, which broadened the JD-R model by investigating the combination effect of job related demand and work resources. Long working hours and ERI put pressure on village doctors [42]. These pressures and low substitutability make highly demanding doctors continue working even while sick [43]. Most studies have shown that being in an effort-reward balance usually leads to fewer depressive symptoms and less presenteeism behavior [22, 23]. The present study also found that village doctors exposed to ERI were at increased risk of depressive symptoms, which is consistent with previous findings [44]. Moreover, our findings further examined that ERI not only strengthened the negative effect of long working hours on depressive symptoms but also strengthened the indirect effect of long working hours on presenteeism by increasing depressive symptoms. Thus, in this study, the direct or indirect effect of long working hours and depressive symptoms on presenteeism was highly associated, and ERI strengthened these associations among Chinese village doctors. These findings provide evidence that more assistance and intervention measures for Chinese village doctors, as well as paying more attention to their daily work and mental health, could attenuate the negative effect of job-related demands on their physical and psychological well-being.

### Strengths and limitations

The present study has three strengths. First, this is the first study to investigate the mediating role of depressive symptoms and the moderating role of ERI in the relationship between long working hours and presenteeism among Chinese village doctors. Second, psychological and work-related variables among village doctors were measured using well-validated tools. Finally, this study was conducted on village doctors in rural clinics, and the response rate was relatively high.

The limitations of the current study should be mentioned. First, a cross-sectional survey does not provide conclusions about the causal mediation or the ability to examine the conceptual model among long working hours, ERI, depressive symptoms, and presenteeism. Although the direct and indirect effects of long working hours on presenteeism via depressive symptoms were noted, this causal study is a statistical convention and should not be interpreted to indicate that cross-sectional analysis can address causality problems. Second, GHQ-12 was used to assess the prevalence of depressive symptoms among village doctors. Although GHQ-12 is a relatively mature general screening scale for mental disorders, there is no consensus on its optimal factor structure, and its four options are a little subjective, so further research is needed. Finally, this study was conducted on village doctors in a province of China, which may decrease the representativeness of the sample. While it may limit the generalizability of the current results, it provides key insights into occupational groups that are less cared for and have a high prevalence of mental health issues.

### Implications and future directions

The findings of this study have important theoretical and practical implications for the scientific prevention and intervention of mental health and presenteeism among Chinese village doctors. Firstly, this study contributes to the literature by providing the high prevalence of depressive symptoms and presenteeism among Chinese village doctors. Chinese healthcare administrators should be more concerned about the deteriorating psychological well-being of village doctors and assess their psychological well-being status regularly. Second, we found a significant effect of long working hours on presenteeism. Organizations should be aware of the potential negative effect of long working hours on the mental health of the village doctors and arrange their working hours reasonably and effectively. Third, the mediating effect of depressive symptoms on the association between long working hours and presenteeism suggests that long working hours and depressive symptoms must be considered concurrently to prevent presenteeism behavior. Therefore, organizations should measure the depressive symptoms and presenteeism behavior of the village doctors regularly. Finally, our study confirmed the negative effects of ERI on working hours, depressive symptoms, and presenteeism, which provides a theoretical contribution to the effort-reward model. It is essential to appropriately decrease working hours and workload to improve the quality of health services and reduce counterproductive behaviors (presenteeism) among village doctors in rural China. For example, managers should foster a supportive environment for village doctors by allocating suitable workloads, reducing red tape, and promoting job security [45]. Furthermore, governments must assure stronger compensation schemes for village doctors and plans that consider the broader psychosocial needs of village doctors due to the high degree of mismatch between perceived effort expended and incentives received.

## Conclusion

Working long hours among village doctors is a very common phenomenon in China. However, there is a growing discontent toward working long hours, and it is becoming increasingly challenging for individuals to maintain their physical and mental health. This study provides evidence for the hypothesis that depressive symptoms mediated the detrimental effect of long working hours on presenteeism of village doctors and examined how ERI strengthened this harmful effect on individuals’ presenteeism by increasing their depressive symptoms.

## Data Availability

The data in this paper are from a field questionnaire survey. The data may be accessed after obtaining the author’s consent (email: yxgao@ntu.edu.cn).

## Abbreviations

ERI: Effort-reward Imbalance
